# Evaluating the use of fluorescent imaging for the quantification of dental fluorosis

**DOI:** 10.1186/1472-6831-12-47

**Published:** 2012-11-01

**Authors:** Michael G McGrady, Roger P Ellwood, Andrew Taylor, Anne Maguire, Michaela Goodwin, Nicola Boothman, Iain A Pretty

**Affiliations:** 1 School of Dentistry, University of Manchester, Manchester, England, M13 9PL, UK; 2 Colgate Palmolive Dental Health Unit, Williams House, Lloyd Street North, M15 6SE, Manchester, England, UK; 3 School of Dental Sciences, University of Newcastle, Newcastle, NE2 4BW, England, UK

## Abstract

**Background:**

The quantification of fluorosis using fluorescence imaging (QLF) hardware and stain analysis software has been demonstrated in selected populations with good correlation between fluorescent image metrics and TF Index scores from photographs. The aim of this study was to evaluate the ability of QLF to quantify fluorosis in a population of subjects (aged 11–13) participating in an epidemiological caries and fluorosis survey in fluoridated and non-fluoridated communities in Northern England.

**Methods:**

Fluorescent images of the maxillary incisors were captured together with standardized photographs were scored blind for fluorosis using the TF Index. Subjects were excluded from the analysis if there were restorations or caries on the maxillary central incisors.

**Results:**

Data were available for 1774 subjects (n=905 Newcastle, n=869 Manchester). The data from the fluorescence method demonstrated a significant correlation with TF Index scores from photographs (Kendall’s tau = 0.332 p<0.0001). However, a number of additional confounding factors such as the presence of extrinsic stain or increased enamel translucency on some subjects without fluorosis or at low levels of fluorosis severity had an adverse impact on tooth fluorescence and hence the outcome variable. This in conjunction with an uneven distribution of subjects across the range of fluorosis presentations may have resulted in the lower than anticipated correlations between the fluorescent imaging metrics and the photographic fluorosis scores. Nevertheless, the fluorescence imaging technique was able to discriminate between a fluoridated and non-fluoridated population (p<0.001).

**Conclusions:**

Despite confounding factors the fluorescence imaging system may provide a useful objective, blinded system for the assessment of enamel fluorosis when used adjunctively with photographic scoring.

## Background

The latter half of the 20th Century demonstrated a decline in the prevalence of dental caries through the use of optimally fluoridated community water supplies and fluoridated oral care products. However, this reduction in caries has also been associated with concerns regarding increased prevalence of dental fluorosis in both fluoridated and non-fluoridated communities [1-4].

In the UK, a systematic review commissioned by the government known as the York Report [5] set out to review the safety and efficacy of water fluoridation. The report stated the occurrence of fluorosis at water fluoride levels of 1ppm was found to be high (predicted 48%, 95% CI 40 to 57). Of this fluorosis, the proportion considered to be aesthetically objectionable was lower (predicted 12.5%, 95% CI 7.0 to 21.5). A study conducted in Newcastle upon Tyne (fluoridated) and Northumberland (non-fluoridated) found increased prevalence of fluorosis in the fluoridated area compared to the non-fluoridated area with similar figures for overall fluorosis prevalence quoted in the York Report but the prevalence of aesthetically objectionable fluorosis was lower at 3.4% [6]. The authors suggested reasons for similarities and differences in prevalence data from other studies [7,8].

There are several possible explanations for the perceived increase in fluorosis prevalence. There could be a true increase in prevalence reflecting an increase in fluoride exposure from various sources of fluoride and an associated increased risk of fluorosis [9]. However, there are other plausible explanations that could explain the increase in prevalence. Traditionally, fluorosis has been assessed by the use of clinical indices such as Dean’s Index [10] and the Thylstrup & Fejerskov (TF) Index [11]. The employment of clinical indices relies upon subjective assessment and interpretation of predetermined criteria, which may impart bias. In light of this and despite a wealth of historical data there have been criticisms of the use of clinical indices in the York Report and elsewhere in the literature [12,13].

The choice of index may influence the investigation of fluorosis prevalence. Large volumes of data were collected through the work of H Trendley Dean utilizing an index that bore his name. This work subsequently led to the implementation of water fluoridation schemes [10,14-16]. Despite criticism of Dean’s Index [13,17] it remains a popular index particularly in the United States. A major difference between Dean’s Index and the TF Index is Dean’s Index assesses teeth wetted by saliva and TF Index requires the drying of teeth prior to assessment. The latter technique highlights the presence of more mild presentations of fluorosis which in itself may result in an apparent increase in fluorosis prevalence and difficulties particularly when comparisons are made to historical data using alternate indices [18].

An additional issue with clinical indices is the possibility of examiner bias. This may manifest through lack of blinding during assessment or variability in inter and intra-examiner agreement. There is also a phenomenon of personal thresholding particularly at low levels of fluorosis severity with differences in the application of diagnostic criteria [18,19]. Attempts have been made to address some of the issues associated with the use of clinical indices. The remote scoring of standardized clinical photographs addresses issues pertaining to examiner blinding and facilitates the longitudinal assessment of fluorosis through the archiving of materials and repeatability of image capture [20,21]. However, as this technique still fundamentally relies upon an examiner employing a subjective index, all of the confounding issues of a clinical index cannot be overcome. Consensus scoring of remote images may address some issues relating to personal thresholding. A further consideration of the remote scoring technique is the viewing medium for image scoring. Magnification of images may increase the detection of milder forms of fluorosis and hence affect prevalence data relative to historical data and potential prospective data if viewing conditions are not carefully controlled.

The York Report and a report from the Medical Research Council that followed [22] both stated the evidence base of studies on water fluoridation required improvement and were critical of the use of such subjective indices for the assessment of fluorosis. Future work should consider more reliable and objective means of quantifying fluorosis severity and for longitudinal monitoring.

Recent years have seen an emphasis on the detection and quantification of dental caries utilizing emerging technologies and diagnostic sciences [23]. The development of caries detection systems with improved sensitivity and specificity over traditional visual and tactile techniques has invigorated the field of cariology enabling more preventative interventions to be used more successfully in preventing caries and the remineralization of early carious lesions. Unfortunately, the advances within cariology have not been reflected in the study of fluorosis where clinical indices still remain the gold standard. However, consideration has been made in the literature to the application of optical techniques employed in caries detection for assessment of fluorosis [12]. One such technique is quantitative light induced fluorescence (QLF). QLF has been investigated as a means of detecting and quantifying early enamel carious lesions [24,25] and has since be explored as a tool for quantifying dental plaque, tooth surface loss (erosion), extrinsic stain and for the quantification of fluorosis [26-29].

Early work on the use of QLF in fluorosis quantification was encouraging [28]. A novel software analysis technique was designed to overcome the difference in presentation of caries (discreet lesions) and fluorosis (diffuse lesions) and the resultant differences in fluorescence signal when using fluorescent imaging. On a selected population with milder forms of fluorosis, QLF achieved very good intra class correlation coefficients (ICC) when compared to the TF Index (Kendall’s Tau = 0.869). However, there were a number of confounding factors. There is an inherent difficulty in determining the potential of QLF as a means of quantifying fluorosis as there is no current acceptable gold standard with which to compare the output metrics of a fluorescent imaging system. The ordinal data derived from a subjective clinical index cannot be easily compared to the continuous data generated from QLF. Hence the analysis could only determine the association between the two techniques, not true agreement. Furthermore, as QLF relies upon the detection of changes in fluorescence between “sound” and “unsound” enamel, any artefact-inducing scattering of the reflected light from the tooth surface could result in a change in fluorescence and aberrant readings for fluorosis quantification. Such artefacts include presence of caries, extrinsic stain, restorations and non-fluorotic opacities. Nevertheless, QLF demonstrated in a small, selected population with a relatively limited range of fluorosis presentations the potential as a means of delivering a system for the reliable, objective quantification of enamel fluorosis.

Subsequent work aimed not only to refine the QLF system in fluorosis quantification by investigating alternate analysis techniques but also determining if QLF could discriminate between a wider range of presentations of fluorosis severity in larger populations with varying exposures to fluoride [30]. The outcome of this work determined the use of a convex hull software algorithm was a more reliable means of quantifying fluorosis and that QLF could discriminate between populations with differing fluoride exposure and fluorosis severity. However, the confounding factors remained unresolved. Despite these limitations QLF still demonstrated potential as a means of objective, blinded quantification and a means of providing a system for longitudinal monitoring.

The aim of this study was to evaluate the use of fluorescent imaging for the quantification of dental fluorosis in an epidemiological survey and to determine the level of association with remote photographic scoring using a standard clinical index.

## Methods

Subjects were selected for this study had participated in an epidemiological survey looking at caries and fluorosis prevalence and severity in two areas in Northern England, Newcastle upon Tyne which is has community water supplies fluoridated at an adjusted level of 1 mgF/L and Greater Manchester which receives non-fluoridated water supplies. The protocol for the study received ethical approval from the University of Manchester Committee on Ethics on Research on Human Beings (ref: 07952).

### Screening and selection of participants

Subjects were healthy males and females aged 11–13 years old who were lifetime residents of their locality. Written consent was obtained from the subjects after the parents or carers had been given two opportunities to object to their child’s participation via a postal return of pre-prepared forms sent out prior to study recruitment.

**Figure 1:**
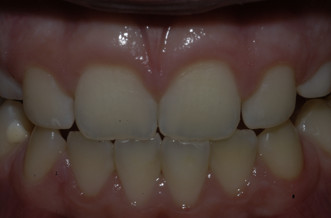
Photographic image of maxillary incisors using standardized technique.

Consented subjects were assigned a five-digit subject ID number based on the sequence of their recruitment. During the observational survey all subjects had standardized conventional digital photographs taken of the maxillary central incisors [31] after the teeth had been cleaned and dried for one minute with cotton wool rolls (Figure 1). The images were exported to a computer and scored for fluorosis using the Thystrup & Fejerskov (TF) Index by a trained examiner (MGM) based at a remote location. The images were presented in a randomized and blind manner in order to ensure the examiner was unaware of the participant’s residential status and fluoride content of community water supply.

### Fluorescence image capture

**Figure 2:**
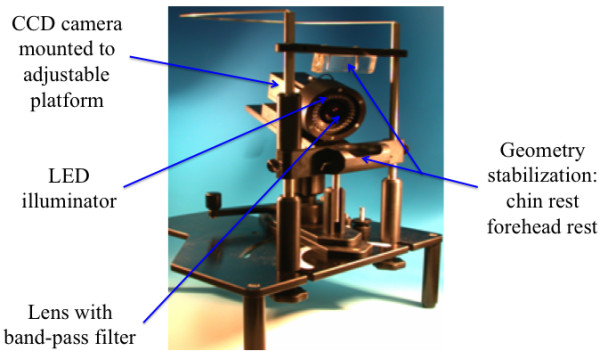
Image of bespoke QLF array together with geometry stabilizing equipment.

**Figure 3:**
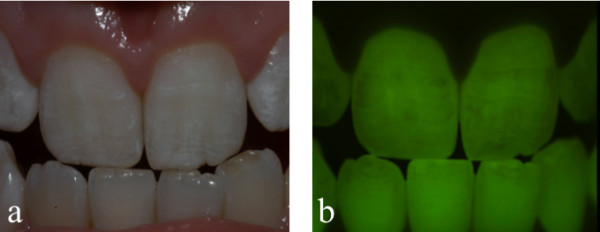
Images of a subject with mild fluorosis (TF2): a) standardized digital image of maxillary central incisors; b) image generated by QLF – darker areas depicting loss of fluorescence (enamel fluorosis).

The imaging equipment comprised a custom-built stabilizing unit, comprising an adjustable head and chin support and a camera focus platform connected to a high-resolution 3 CCD camera (Jai M91P, Jai Corp., Copenhagen, Denmark) and illuminator (a custom made LED array with variable illumination emitting light with peak source at 405-nm). The platform enabled the camera to be repositioned and focussed while the subject remained static (Figure 2). Typical images generated for a subject with mild fluorosis (TF2) are illustrated in Figure 3. Areas of the teeth that are seen as bright green under QLF depict regions of sound, or unaffected enamel. Where there has been a disturbance in enamel mineralization (such as fluorosis) the resulting areas of hypomineralization results in a reduction, or loss, or fluorescence and is seen as darker areas when viewed with QLF. Images captured by QLF can be analysed using software algorithms to produce metrics relating to the fraction of tooth area considered to be fluorotic (Area_ch_), the average fluorescence loss of tooth area considered to be fluorotic (ΔF_ch_) and the average fluorescence loss over entire tooth surface (ΔQ_ch_).

### Software

A convex hull analysis software package originally designed to quantify stain on teeth was utilized [29]. The software was designed to detect diffuse areas on the tooth surface using an algorithm based on a convex hull to detect and quantify the diffuse areas of hypomineralization associated with fluorosis. The application of this methodology has been described in the literature [30].

### Data management and analysis

The data for the photographic TF index scores from the epidemiological survey were entered into the Statistical Package for Social Sciences (SPSS 16.0) along with the metrics from the analysis of the fluorescent images using convex hull software. For each subject, the higher of the two scores on the maxillary central incisors was used in the statistical analysis. Correlation coefficients between the photographic scores and the output from the software analyses were determined using for comparison with the metrics of ΔF_ch_, Area_ch_ and ΔQ_ch_.

## Results

Once data cleaning had been completed data were available for 1774 (Newcastle 905, Manchester 869) subjects with QLF images of the maxillary central incisors and corresponding photographic fluorosis scores using TF index. This data is presented in Table 1 demonstrating frequency counts for fluorosis severity. As dental fluorosis is not endemic in the UK, the data did not present a uniform distribution of presentations of severity, with 59% of the patients not having the condition and 32% of subjects having fluorosis with a severity of TF1 when assessed by photographic scoring using a standard clinical index. The data were analysed to determine the association between the photographic scores and the QLF metrics. The data demonstrated an increase in mean value for each QLF metric as the fluorosis severity increased (Table 2). Intra class correlation coefficients were calculated for each of the QLF output metrics (ΔF_ch_, Area_ch_ and ΔQ_ch_) and are described in Table 2. Each of the QLF metrics demonstrated significant associations with the photographic scores for fluorosis with Kendall’s tau values of 0.342, 0.282 and 0.332 for area, ΔF_ch_ and ΔQ_ch_ respectively. The metric for Area_ch_ had the highest association with the photographic scores, but in terms of fluorosis quantification, the QLF metric for ΔQ_ch_ holds the most relevance, as it is a composite of the degree of fluorescence loss and a measure of the area of tooth surface involved.

**Table 1:** Frequency counts of subjects at each level of TF Index score

**Photographic TF Score**	**City (frequency counts)**				**Total**
	**Newcastle**		**Manchester**		
	**n**	**%**	**n**	**%**	
0	409	45%	638	73%	1047
1	355	39%	209	24%	564
2	79	9%	16	2%	95
3	53	6%	4	1%	57
4	8	1%	0	0%	8
5	1	0.1%	2	0.2%	3
Total	905		869		1774

**Table 2:** Intra class correlation coefficients for QLF metrics and mean metric values for each TF index score

**QLF mETRIC (mean)**	**TF SCORE**						**Spearman’s rho**	**Kendall’s tau b**
	**0**	**1**	**2**	**3**	**4**	**5**	**P<0.0001**	
**Area**_**ch**_	0.070	0.097	0.177	0.248	0.317	0.402	.421	.342
**ΔF**_**ch**_	0.043	0.047	0.058	0.070	0.086	0.108	.349	.282
**ΔQ**_**ch**_	0.004	0.005	0.011	0.018	0.034	0.046	.410	.332

**Figure 4:**
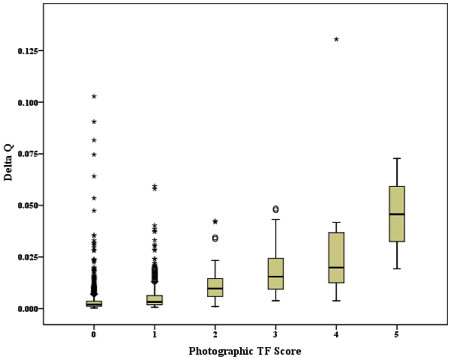
**Boxplot of QLF metric for Delta Q**_**ch**_**against photographic TF Index score (with subject outliers).**

A boxplot of ΔQ_ch_ against TF score (Figure 4) demonstrates the increase in magnitude of the QLF metric as the fluorosis severity increases. It also revealed a large number of outliers in the dataset particularly for lower severities of fluorosis. Outliers were identified and the QLF images and photographs for these subjects re-examined to find possible explanations for the results. The presence of caries, restorations and demarcated opacities are known to be confounders for QLF and most outliers were found to demonstrate one or more of these characteristics. A summary of additional confounding factors and the associated frequency counts from subjects with TF0 and TF1 are shown in Table 3. The presence of extrinsic stain was the most common additional confounding factor identified (16 subjects) but there were more subjects (30) where no plausible explanation for the QLF outcome could be provided.

**Table 3:** Description and frequency of subjects with additional compounding factors

**Confounding factor**	**Number of subjects**	
	**TF0**	**TF1**
Extrinsic stain	13	3
Enamel erosion	1	-
Translucent enamel	2	-
Enamel fractures	2	-
Missed demarcated opacity	3	7
Unknown	14	16
**Total**	**35**	**26**

The data was then examined to determine if the two populations (fluoridated and non-fluoridated) could be separated for fluorosis prevalence using the fluorescent imaging technique. Ranks and sum of ranks for each QLF metric were calculated for both cities and are displayed in Table 4. Non-parametric analysis using Man Whitney U tests demonstrated significant differences between the fluoridated and non-fluoridated population for each of the QLF metrics (p<0.001).

**Table 4:** Comparison of QLF metrics between cities

**QLF metric**	**City**	**Mean rank**	**Sum of ranks**	**Mann Whitney U**	**Sig (2-tailed)**
**Area**_**ch**_	Newcastle (N=905)	1014.67	918274.00	278136.00	P<0.001
	Manchester (N=869)	755.06	656151.00		
**ΔF**_**ch**_	Newcastle (N=905)	976.62	883843.00	312576.00	P<0.001
	Manchester (N=869)	794.69	690582.00		
**ΔQ**_**ch**_	Newcastle (N=905)	1006.98	911320.00	285090.00	P<0.001
	Manchester (N=869)	763.07	663105.00		

**Figure 5:**
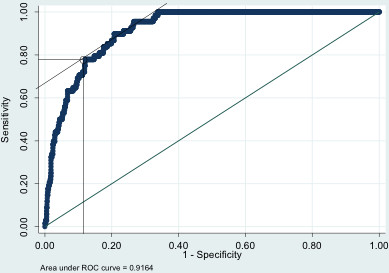
ROC curve for QLF fluorosis detection.

The data was exported to Stata (release 11, StataCorp, TX, USA) and a receiver operating characteristic (ROC) curve produced using a classification model for the QLF metric output ΔQ_ch_ and a classifier boundary, or threshold, for fluorosis (TF photo score) of ≤2 and ≥3. The ROC curve is illustrated in Figure 5. The Area under the curve (AUC) was 0.9164 suggesting an excellent level of accuracy.

Contingency tables for subjects with or without fluorosis for the QLF metric ΔQ_ch_ and photographic TF scores ≤2 and ≥3 are shown in Table 5. Both methodologies demonstrate differences between the fluoridated and non- fluoridated populations. The proportion of subjects with fluorosis differed between the two methodologies. The proportion for photographic scores was 1% in Manchester and 7% Newcastle, whereas for ΔQ_ch_ the proportions were 10% and 19% in Manchester and Newcastle respectively. The results suggested the QLF technique was able to differentiate between the fluoridated and non-fluoridated populations. However, whilst the direction of difference was the same the difference in magnitude of the proportions between the two methodologies highlighted issues relating to the sensitivity and specificity of fluorosis detection.

**Table 5:** Contingency table of subjects with and without fluorosis as determined by Δ Q (QLF) and photographic TF score

	**Condition**	**Manchester (869)**	**Newcastle (905)**	**χ**^**2**^**chi square**
**Fluorosis ΔQ**_**ch**_	No Fluorosis	783 (90%)	731 (81%)	χ^2^ (1)= 31.735, P<0.0001
	Fluorosis	86 (10%)	172 (19%)	
**Fluorosis photo**	Fluorosis TF 0-2	863 (99%)	843 (93%)	χ^2^ (1)= 45.640, P<0.0001
	Fluorosis TF 3-5	6 (1%)	62 (7%)	

## Discussion

The purpose of this study was to use the QLF system within a standard epidemiological survey. The earlier work of Pretty et al. [28] and use in larger populations [30] identified strengths and weaknesses of fluorosis quantification by fluorescent imaging techniques. The encouraging results from the early work on intra class correlations between the QLF metrics and TF scores and the ability to detect differences in populations with different fluoride exposures gave justification for incorporating the system into an epidemiological survey. However, many of the issues raised by Pretty et al. [28] remained unresolved.

The lack of an appropriate gold standard for comparison with the QLF metrics gave rise to statistical and interpretive problems as the data from the TF index is on an ordinal scale from 0 to 9 whereas the output from the QLF metrics generates continuous data. The consequence is there are no appropriate statistical methods to assess agreement. Hence, the use of correlations during analysis demonstrates the association between the outcomes, which should not be interpreted as agreement.

Choice of gold standard is not a unique issue to fluorosis quantification. QLF and other fluorescent imaging techniques have been used to detect caries with similar issues regarding agreement between outcome measures [32]. In the case of caries detection, gold standards exist through histological examination using light microscopy and microradiography. These techniques have enabled the development of more robust assessment of agreement with QLF metrics relating to caries detection [33] with cut off thresholds for the fluorescence devices. The validation of such devices for caries detection is an evolving subject influenced by the tooth surface under investigation and has been facilitated by the existence of more appropriate gold standards. The absence of an appropriate gold standard for fluorosis quantification resulted in a cut off threshold for ΔQ_ch_ being determined from the ROC curve. This should not be interpreted as a transferable threshold for QLF analysis of other populations as it was not validated.

The decision to use the TF Index for fluorosis scoring was influenced by the index being based on the histological features associated with the presentation of the condition [11]. However, fundamental differences exist between the aspects of the condition assessed by QLF and the TF Index. The former detects fluorosis over the whole tooth surface through fluorescence loss in image pixels whereas the latter assesses fluorosis not only from the clinical manifestations of histological changes but also from the patterns of presentation such as diffuse lines and confluent areas. Hence, the TF Index has no direct means of assessing the area of tooth surface involved. It is therefore interesting to find from the results of this study the QLF metric for area has the strongest correlation with the TF scores.

An inherent limitation of QLF is the inability to differentiate fluorescence loss as a result of fluorosis; other forms of developmental enamel defects and tooth surface phenomena such as enamel fractures and extrinsic stain. There is evidence to suggest that the use of computer software techniques may facilitate this process [34] but this would involve more complicated image processing and tooth mapping prior to analysis.

## Conclusions

The results of this study suggest that QLF has the ability to reliably quantify fluorosis in an epidemiological setting, albeit assisted by clinical diagnosis. In addition, QLF was able to discriminate between fluoridated and non-fluoridated populations. The intra class correlation coefficients are lower than those previously obtained [28,30]. However, these associations are still significant and it should be stated that through each iterative stage of QLF evaluation the study populations have become larger, less selected, have demonstrated greater variety of fluorosis presentation and the potential for more confounding factors. Improved image mapping and software analysis may reduce these phenomena. Fluorescent imaging techniques such as QLF appear to have a high sensitivity but reduced specificity when employed in the detection and quantification of fluorosis impacting on the potential for these technologies to act as diagnostic tools for this condition. However, despite these limitations, QLF has the potential to monitor fluorosis longitudinally at both a population and individual level. Despite confounding factors the fluorescence imaging system may provide a useful objective, blinded system for the assessment of enamel fluorosis when used adjunctively with photographic scoring.

## Abbreviations

AUC: Area under the curve; CCD: Charge-coupled device; ICC: Intra-class correlation coefficients; LED: Light emitting diode; QLF: Quantitative light-induced fluorescence; ROC: Receiver operating characteristics; SPSS: Statistical package for social sciences; TF: Thylstrup & fejerskov index; ΔF_ch_: Average fluorescence loss of areas considered fluorosis (convex hull technique); ΔQ_ch_: Average fluorescence loss over entire tooth surface.

## Competing interests

None of the authors are aware of any competing interests in the production of this manuscript.

## Authors’ contributions

MGM prepared the protocol, conducted the fieldwork, was involved in the analysis of data and wrote the manuscript. RPE provided input into the study design and the manuscript. AM acted as local Investigator and provided input into study design and he manuscript. NB co-ordinated the study, conducted fieldwork and participant instruction on questionnaire completion. MG conducted the statistical analysis. IAP was Principal Investigator and provided input into study design and manuscript. All authors read and approved the final manuscript.

## Pre-publication history

The pre-publication history for this paper can be accessed here:

http://www.biomedcentral.com/1472-6831/12/47/prepub
